# Estimating signal and noise of time-resolved X-ray solution scattering data at synchrotrons and XFELs

**DOI:** 10.1107/S1600577520002738

**Published:** 2020-03-31

**Authors:** Jungmin Kim, Jong Goo Kim, Hosung Ki, Chi Woo Ahn, Hyotcherl Ihee

**Affiliations:** a Department of Chemistry and KI for the BioCentury, Korea Advanced Institute of Science and Technology (KAIST), 291 Daehak-ro, Yuseong-gu, Daejeon 34141, Republic of Korea; b Center for Nanomaterials and Chemical Reactions, Institute for Basic Science (IBS), 291 Daehak-ro, Yuseong-gu, Daejeon 34141, Republic of Korea

**Keywords:** time-resolved X-ray solution scattering, time-resolved X-ray liquidography, simulation, signal-to-noise ratio, solvent cage

## Abstract

Elucidating the structural dynamics of small molecules and proteins in the liquid solution phase is essential to ensure a fundamental understanding of their reaction mechanisms. In this regard, time-resolved X-ray solution scattering (TRXSS), also known as time-resolved X-ray liquidography (TRXL), has been established as a powerful technique for obtaining the structural information of reaction intermediates and products in the liquid solution phase and is expected to be applied to a wider range of molecules in the future. A TRXL experiment is generally performed at the beamline of a synchrotron or an X-ray free-electron laser (XFEL) to provide intense and short X-ray pulses. Considering the limited opportunities to use these facilities, it is necessary to verify the plausibility of a target experiment prior to the actual experiment. For this purpose, a program has been developed, referred to as *S-cube*, which is short for a *Solution Scattering Simulator*. This code allows the routine estimation of the shape and signal-to-noise ratio (SNR) of TRXL data from known experimental parameters. Specifically, *S-cube* calculates the difference scattering curve and the associated quantum noise on the basis of the molecular structure of the target reactant and product, the target solvent, the energy of the pump laser pulse and the specifications of the beamline to be used. Employing a simplified form for the pair-distribution function required to calculate the solute–solvent cross term greatly increases the calculation speed as compared with a typical TRXL data analysis. Demonstrative applications of *S-cube* are presented, including the estimation of the expected TRXL data and SNR level for the future LCLS-II HE beamlines.

## Abstract 2

The simulation of a signal and signal-to-noice ratio for time-resolved X-ray solution scattering data is presented.

## 1. Introduction   

Understanding the solution-phase reaction mechanism is central to the field of chemistry. Fast processes in solutions involving short-lived intermediates are often investigated by time-resolved spectroscopy, but the associated molecular structural changes in general are not direct observables of time-resolved spectroscopy, which relies on the transitions between energy states. In this regard, time-resolved X-ray solution scattering (TRXSS), also known as time-resolved X-ray liquidography (TRXL), has been established as a powerful tool for studying molecular structural dynamics in the liquid solution phase (Kjaer *et al.*, 2019 ▸; Haldrup *et al.*, 2012 ▸, 2019 ▸; Salassa *et al.*, 2010 ▸; Kim *et al.*, 2012 ▸; Ahn *et al.*, 2018 ▸; Biasin *et al.*, 2018 ▸; Canton *et al.*, 2015 ▸; Kim, Kim, *et al.*, 2016 ▸; Kong *et al.*, 2019 ▸; Leshchev *et al.*, 2018 ▸; Berntsson *et al.*, 2017 ▸; Josts *et al.*, 2018 ▸; Kim, Yang, *et al.*, 2016 ▸; Kim, Ganesan, *et al.*, 2018 ▸; Rimmerman *et al.*, 2017 ▸; Arnlund *et al.*, 2014 ▸; Kim, Muniyappan, *et al.*, 2016 ▸; Malmerberg *et al.*, 2015 ▸; Haldrup *et al.*, 2009 ▸; Christensen *et al.*, 2009 ▸; Cammarata *et al.*, 2006 ▸, 2008 ▸; Kong *et al.*, 2007 ▸; Ihee *et al.*, 2005 ▸; Plech *et al.*, 2004 ▸). In a typical experiment, an optical laser pulse is used to initiate a reaction and after a time delay an X-ray pulse is sent to probe the reaction progress via X-ray scattering. TRXL has been applied to a wide range of molecules ranging from small molecules such as organometallic compounds (Biasin *et al.*, 2018 ▸; Kim, Kim, *et al.*, 2016 ▸; Kong *et al.*, 2019 ▸; Leshchev *et al.*, 2018 ▸; Canton *et al.*, 2015 ▸; Haldrup *et al.*, 2012 ▸; Salassa *et al.*, 2010 ▸; Haldrup *et al.*, 2019 ▸; Kjaer *et al.*, 2019 ▸; Kong *et al.*, 2007 ▸) and hydro­carbons (Ahn *et al.*, 2018 ▸; Kim *et al.*, 2012 ▸; Ihee *et al.*, 2005 ▸; Davidsson *et al.*, 2005 ▸) to macromolecules such as proteins (Berntsson *et al.*, 2017 ▸; Josts *et al.*, 2018 ▸; Kim, Yang, *et al.*, 2016 ▸; Kim, Ganesan, *et al.*, 2018 ▸; Rimmerman *et al.*, 2017 ▸; Arnlund *et al.*, 2014 ▸; Kim, Muniyappan, *et al.*, 2016 ▸; Malmerberg *et al.*, 2011 ▸, 2015 ▸; Konuma *et al.*, 2011 ▸; Westenhoff *et al.*, 2010 ▸; Cho *et al.*, 2010 ▸; Andersson *et al.*, 2009 ▸; Cammarata *et al.*, 2008 ▸). Due to the successful applications of TRXL to a wide range of molecules and reactions, applications of TRXL are expected to increase in the future.

Nevertheless, TRXL is not an omnipotent technique, and it has two major limitations. The first is the weak sensitivity to the solute compared with time-resolved spectroscopy and the second is the limited number of beamlines for TRXL. Generally, the scattering signal from the solute is weaker than the spectroscopic signal in time-resolved spectroscopy because the total scattering signal is dominated by the solvent, which generally exists in greater amounts than the solute and usually does not participate in the net reaction (Neutze *et al.*, 2001 ▸; Plech *et al.*, 2004 ▸; Kim *et al.*, 2009 ▸; Haldrup *et al.*, 2010 ▸). Due to the relatively weak signal, it is necessary to use extremely intense X-ray pulses to collect data successfully. Accordingly, TRXL experiments are conducted at large-scale facilities that can produce such intense X-ray pulses, such as third-generation synchrotrons, which offer approximately 100 ps-long X-ray pulses, or at X-ray free-electron lasers (XFELs), which provide sub-100 fs-long X-ray pulses. Access to these facilities is generally competitive.

**Figure 1:**
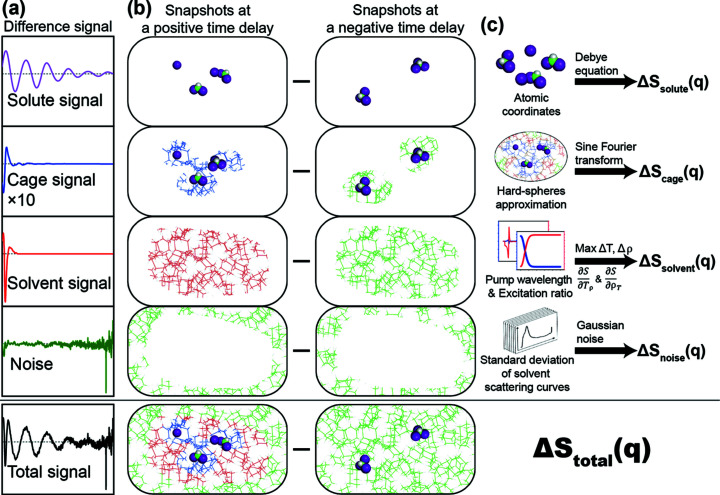
The four signals [solute (solute-only), cage (solute–solvent cross), solvent (solvent-only) and noise] that comprise the difference signal are schematically illustrated. (*a*) The difference curves corresponding to the four contributing signals and the total signal. (*b*) Snapshots of solute and solvent molecules for each signal. The snapshot for the solute signal represents the structure change of the solute molecules induced by a pump pulse. The snapshot for the cage signal represents the structure change of the solvent molecules surrounding the solute caused by the structural change of the solute molecules. The snapshot for the solvent signal represents the structural changes due to temperature and density changes of the solvent from heating caused by excited solute molecules. (*c*) Schematic diagram of the simulation of each signal in *S-cube*. The solute signal is calculated from the concentration and structure of the reactants and products using the Debye equation. For calculation of the solvent signal, maximum changes of temperature (Δ*T*) and density (Δρ) are calculated from the energy of the pump laser and the number of light-absorbing solute molecules. Subsequently, the solvent signal is obtained from the sum of each product that are maximum Δ*T* × (δ*S*/δT)_ρ_ and Δρ × (δ*S*/δρ)_*T*_. The noise is acquired by considering the simulation environments and σ_solvent_. The cage signal can be calculated using the sine Fourier transform from radial distribution functions of MD simulations or the hard-spheres approximation.

Due to these limitations, it is crucial to estimate the plausibility of a target TRXL experiment theoretically prior to performing the actual experiment. The X-ray scattering signal from a liquid solution sample is composed of four signals; the solute-only signal, the solute–solvent cross signal (cage signal), the solvent-only signal and noise, as illustrated in Fig. 1 ▸. Because the structural dynamics information of a reaction is mostly contained in the solute-only signal, the relative magnitude of the solute-only signal with respect to the total signal is the determining factor for the successful application of TRXL. In addition, if the expected signal level relative to the experimental noise level, that is, the signal-to-noise ratio (SNR), can be predicted prior to the actual beam time, it will allow an estimation of the accumulation time required to obtain a sufficient SNR suitable for the purpose of the experiment. Accordingly, it would greatly facilitate the planning of experiments and increase the success rate.

In this work, we present a program which can aid in the forecasting of the quality of experimental TRXL data. This program, named *S-cube* (*S* ^3^), which is short for a *Solution Scattering Simulator*, is a MATLAB-based graphical user interface (GUI) application. On the basis of the given experimental conditions, *e.g.* the intensity of the X-ray sources, the target solute and solvent, and the data accumulation time, *S-cube* calculates the solute-only signal, the solute–solvent cross signal, the solvent-only signal and the expected noise level for a desired target reaction for investigation. We show how *S-cube* can be used to estimate the plausibility of a TRXL experiment or to design a successful experiment. In particular, we demonstrate how *S-cube* can be used to simulate the signal level for a chemical reaction with a variety of experimental conditions. In addition, we use *S-cube* to show the expected signal level of a TRXL experiment at the upcoming LCLS-II HE beamlines (LCLS, 2018 ▸; LCLS-II, 2018 ▸).

## 2. Results and discussion   

### 2.1. Simulation of the TRXL signal using *S-cube*   

The TRXL signal consists of the solute-only term, solute–solvent cross (cage) term, solvent-only term and noise as shown in Figs. 1(*a*) and 1(*b*) ▸. Therefore, these four terms should be considered in the data simulation. Fig. 1(*c*) ▸ schematically illustrates how each of the four signals can be calculated. In the *S-cube* simulation, the simulated signal [Δ*S*_sim_(*q*)] is obtained by summing the solute-only signal [Δ*S*_solute_(*q*)], the solvent-only signal [Δ*S*_solvent_(*q*)], the solute–solvent cross signal [Δ*S*_cage_(*q*)] and the noise [Δ*S*_noise_(*q*)],
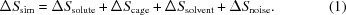
The key aspects of TRXL signal simulation using *S-cube* are as follows: (i) Δ*S*_cage_(*q*) is calculated much more quickly than in a typical program by treating molecules as hard spheres instead of relying on molecular dynamics (MD) simulations, which is the most time-consuming step, and (ii) the expected experimental noise level can be estimated in addition to theoretical signal components of the solute-only, solvent-only and solute–solvent cross signals. Thus, Δ*S*_cage_(*q*) and Δ*S*_noise_(*q*) are discussed in this section, and the details are described in the *Methods* section[Sec sec5] and elsewhere (Neutze *et al.*, 2001 ▸; Plech *et al.*, 2004 ▸; Kim *et al.*, 2009 ▸; Haldrup *et al.*, 2010 ▸).

**Figure 2:**
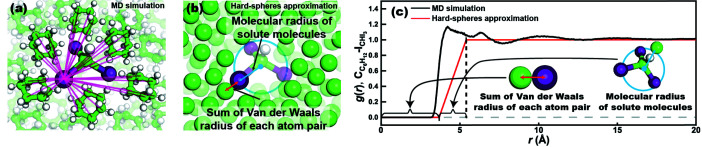
Comparison between the MD simulation and the hard-spheres approximation method for CHI_3_ in cyclo­hexane. (*a*) Schematic diagram for the MD simulation. The transparent magenta lines represent interatomic distances between the iodine atom in CHI_3_ and carbon atoms in cyclo­hexane. (*b*) Schematic diagram for the hard-spheres approximation. (*c*) Comparison of the pair distribution functions, *g*(*r*), between iodine in CHI_3_ and carbon in cyclo­hexane from an MD simulation and the hard-spheres approximation.

**Figure 3:**
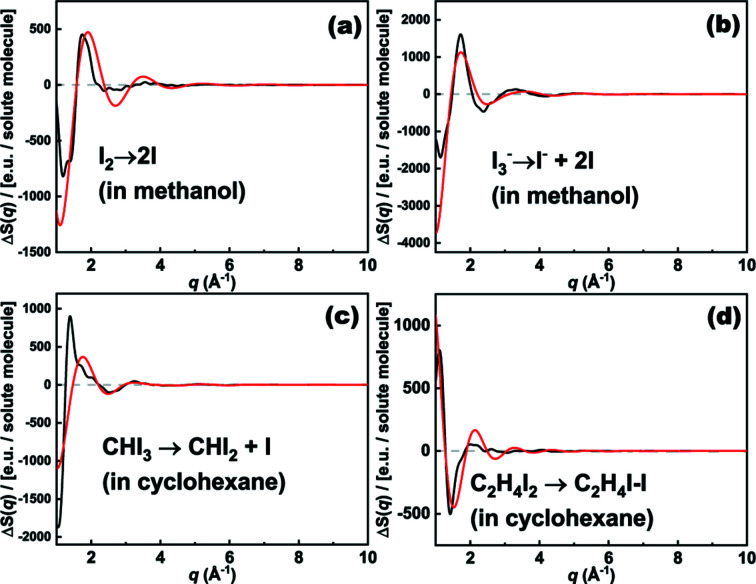
Comparison of solute–solvent cross terms obtained from MD simulations (black curves) and the hard-spheres approximation method (red curves) for various reactions. The difference scattering intensity, Δ*S*(*q*), is for one solute molecule and divided by the scattering intensity of a single electron. As a result, Δ*S*(*q*) is in electron units (e.u.) per solute molecule.

Δ*S*_cage_(*q*) is calculated from the sine Fourier transform of pair-distribution functions (PDFs), *g*(*r*), between atoms in solute and solvent in the following equation (Dohn *et al.*, 2015 ▸; Kim *et al.*, 2009 ▸),
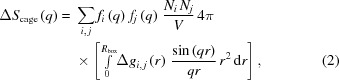
where *i* and *j* are indices of atom types in solute and solvent molecules, respectively, *f*_*i*_ and *f*_*j*_ are atomic form factors for atom-types *i* and *j*, *N*_*i*_ and *N*_*j*_ are the numbers of atoms for atom-type *i* and *j*, and *V* is the box volume for MD simulations. In typical TRXL data analysis, PDFs are obtained from MD simulations [see Fig. 2(*a*) ▸], which demand a large amount of computation. *g*_*ij*_(*r*) is the PDF which is calculated for every pair between types of atom *i* in solute and types of atom *j* in solvent. To substantially reduce the computation time, *S-cube* does not use MD simulations and instead adopts a simplified *g*_*ij*_(*r*) for atom pair *i* in solute and *j* in solvent using a hard-spheres approximation method [see Fig. 2(*b*) ▸]. As shown in Fig. 2(*c*) ▸, the hard-spheres approximation method simplified the *g*_*ij*_(*r*) a trapezoidal function using the following equation (see Fig. 2 ▸),
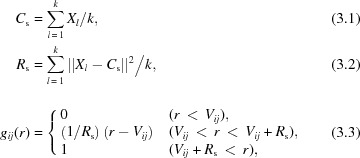
where *V*_*ij*_ is the sum of the van der Waals radii of the *i*th atom and *j*th atoms, and *R*_s_ is the molecular radius of the solute molecule, *C*_s_ is the centroid of the solute molecule, *X*_*l*_ is the position of the *l*th atom in the solute molecule, and *k* is the total number of atoms in the solute molecule. In equation (3.3), the simplified *g*_*ij*_(*r*) is calculated in the following three regimes: (1) when *r* < *V*_*ij*_, *g*_*ij*_(*r*) is set to 0 due to the impenetrability of the hard sphere; (2) when *V*_*ij*_ + *R*_s_ < *r*, the interaction between the solute and solvent is approximated to be zero and thus *g*_*ij*_(*r*) is set to 1; (3) when *V*_*ij*_ < *r* < *V*_*ij*_ + *R*_s_, *g*_*ij*_(*r*) increases linearly from 0 to 1 as a function of *r*. Figs. 3 ▸ and S5 compare Δ*S*_cage_(*q*) calculated from MD simulations and the hard-spheres approximation method for various reactions. Although fine shapes cannot be reproduced by the hard-spheres approximation, the agreement with regard to the *q* range above 1 Å^−1^ in terms of the overall trend and amplitude appears sufficient for the purpose of estimating the contribution of the solute–solvent cross term relative to the other terms.

In a typical data analysis, Δ*S*_noise_(*q*) is not calculated and only the other three signals are calculated to generate the theoretical curve to be compared with the experimental data. Because the purpose of *S-cube* is to predict the plausibility of a target experiment, the estimation of Δ*S*_noise_(*q*) becomes important.

To simulate Δ*S*_noise_ of an experiment, as it is well known that the noise consists of several components having different physical origins, it is indeed ideal to take into account all the components of the noise. The noise components can be classified into two major categories: random noise and systematic noise. Random noise gives stochastic fluctuation of measured intensities around their true values. One of the representative components that consist of random noise is quantum noise. Quantum noise, which is also known as ‘Poisson noise’ or ‘shot noise’, originates from the quantum nature of scattered X-ray photons. Due to the Poisson nature of the noise, the amplitude of quantum noise is determined to be proportional to the square root of the scattering intensity, regardless of the experimental condition. In most cases, the quantum noise dominates the entire experimental noise, except for the two representative cases:

(1) When the scattering signal is too weak so that each detector pixel cannot receive a sufficient number of photons. In this case, readout noise, which emerges from the conversion process of the intensity of incident light to the electric signal, can be the most dominant source of the experimental noise as the amplitude of the quantum noise decreases due to the decrease of the intensity of scattering intensities.

(2) When the readout rate of the detector is too fast. It is known that readout noise of the detector abruptly increases with the increase of the readout rate of the signal when normalized to the data collection time. Accordingly, the readout noise can be the governing component of the entire noise with an experiment with a high readout rate.

Nevertheless, consideration of all of these noise components complicates the simulation algorithm unnecessarily, and thus can potentially confuse the users of the simulation code. Also, in a typical TRXL experiment, the incident photon flux is high and as a result the Poisson noise due to the scattered photons impinging on the detector is larger than those from dark noise and readout noise from the detector by orders of magnitude. For this reason, the *S-cube* simulation only considers the contribution of the Poisson noise when estimating the amplitude of the noise in the experimental data. Therefore, for the simulations in *S-cube*, we allow the user to choose between two different methods of noise estimation. As the first method, which is for a more accurate estimation of the noise, one can collect solvent scattering data under similar experimental conditions to the experiment to be simulated in advance so that *S-cube* can use the obtained solvent data as the reference. Use of the experimental data measured under similar experimental conditions as a reference for the simulation indeed guarantees a precise estimation of the amplitude of the experimental noise as the contributions of all the possible components of the noise are reflected in the real data. Nevertheless, considering the limited accessibility to the X-ray sources for a TRXL experiment, this method is impractical for general users preparing an experiment. Accordingly, we also provide a second, the simpler, but more general method as an option for *S-cube* simulation. In this alternative method, the noise level is estimated based on reference solvent scattering data which are provided by default in *S-cube*. For this estimation, it is assumed that quantum noise, which dominates Δ*S*_noise_ for a typical experimental condition, is considered as the only source of noise and other sources of noise are ignored for the purpose of simplicity, but rough estimation of the quality of the experimental data. This consideration allows to quickly estimate the SNR of an experiment by only considering the intensity of incident X-rays, as proven in the supporting information. Nevertheless, at the current stage, the number of solvent scattering data provided by *S-cube* is not yet enough to cover all the general experimental conditions, and therefore there is room for update. We will continue to add the solvent data for as many different conditions as possible in order to further improve the reliability of the *S-cube* simulation. All the simulations demonstrated in this work were performed using this second method. A more detailed theoretical background for the second method is as follows.

As discussed in the *Methods* section[Sec sec5], the total standard deviation of the scattering intensity, σ_solution_(*q*), can be approximated to be equal to the standard deviation of the solvent, σ_solvent_(*q*), because the number of solvent molecules is high enough to neglect the other two contributions from the solute molecules and cages. In other words, regardless of the type of solute, σ_solution_(*q*) can be approximated by σ_solvent_(*q*) which can be measured from a separate experiment on a neat solvent.

Assuming that each scattering photon is independent and that the variance of the scattering intensity is proportional to the scattering intensity, the variance of the scattering intensity is proportional to the number of incident scattering photons to the sample per scattering curve (*N*). Thus, σ_solvent_(*q*) can be used to estimate the standard deviation of scattering intensity for a simulated condition, σ_solution_(*q*), using *N*_solvent_ and *N*_simulation_ calculated from the experimental parameters of the X-ray pulse repetition rate (*f*), detector exposure time (*D*) and the number of photons per pulse (*n*) using the following equation,
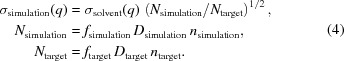

*S-cube* receives the required experimental parameters including σ_solvent_ (including *f*_solvent_, *D*_solvent_ and *n*_solvent_), *f*_target_, *D*_target_ and *n*_target_ as input through the graphical user interface depicted in Fig. S1. Some examples of σ_solvent_(*q*) for experiments conducted at various X-ray facilities are shown in Fig. S2. Meanwhile, scattering photon counting statistics can be assumed to be a Poisson process (Kirian *et al.*, 2011 ▸; Schindler *et al.*, 2016 ▸; Sedlak *et al.*, 2017 ▸), which is popularly chosen to describe the noise of the scattering intensity with a specific standard deviation using Gaussian (Bernadó *et al.*, 2007 ▸; Förster *et al.*, 2008 ▸; Pinfield & Scott, 2014 ▸; Schindler *et al.*, 2016 ▸; Stovgaard *et al.*, 2010 ▸) or Poisson noise (Schneidman-Duhovny *et al.*, 2010 ▸; Sedlak *et al.*, 2017 ▸).

Δ*S*_noise_ is calculated by considering a normal probability distribution which has σ_target_ as its standard deviation as follows,
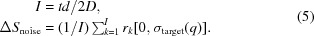
Here, *I* is the number of difference curves for the simulation, *t* is the nominal accumulation time (in seconds), *d* is the duty cycle which is the fraction of the actual accumulation time to the nominal accumulating time (*t*), and *r*_*k*_[0, σ_target_] is the *k*th randomly generated Gaussian noise. The Gaussian noise was generated by sampling random numbers with a normal probability distribution which has zero as the mean value and has σ_target_ as its standard deviation.

### 2.2. Comparison with experimental data   

**Figure 4:**
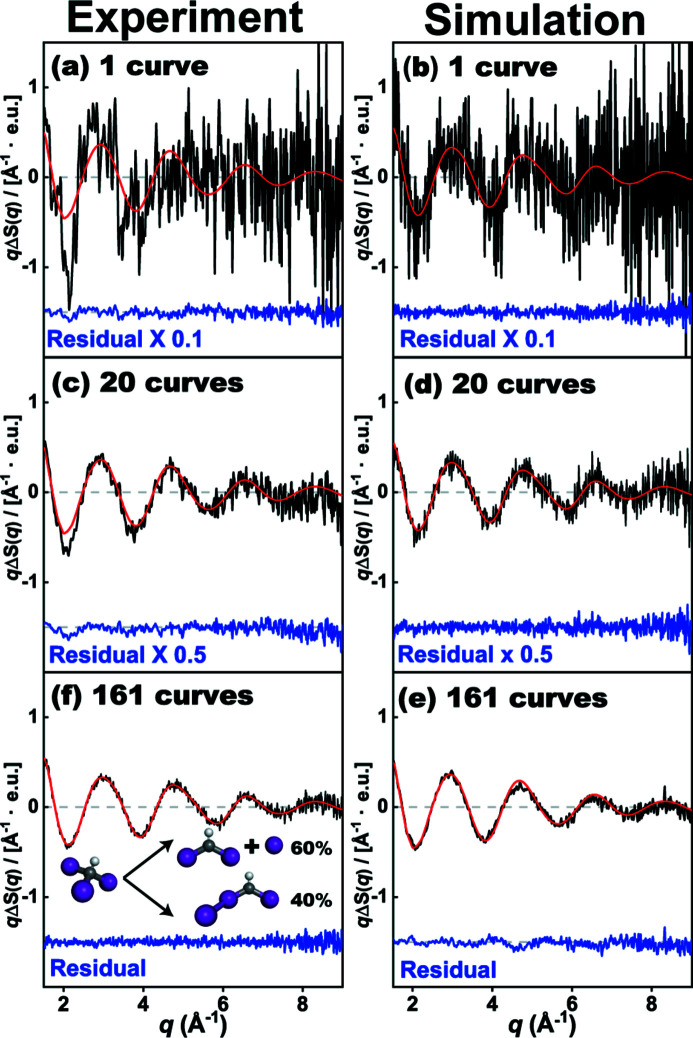
Comparison of experimental (*a*, *c*, *e*) and simulated data (*b*, *d*, *f*) for the difference curve at the time delay of 100 ps for iodo­form dissolved in cyclo­hexane collected at the ID09 beamline of ESRF. The experimental data and simulation results are averaged from one curve (*a*, *b*), 20 curves (*c*, *d*) and 161 curves (*e*, *f*), respectively. In experimental data (*a*, *c*, *e*), the black lines are experimental curves. The red lines are theoretical curves, which are calculated *q*Δ*S*(*q*) from the results reported by Ahn *et al.* (2018 ▸). The blue lines are residual between experimental and theoretical curves. In simulation results (*b*, *d*, *f*), the black, red and blue curves are simulation results with noise, simulation results without noise, and the residuals between simulation results with and without noise, respectively. Note that the residuals in (*a*) and (*b*) are multiplied by 0.1 and those in (*c*) and (*d*) by 0.5. The residuals in experiment and simulation represent noise. The *y*-axis indicates *q*Δ*S*(*q*), the difference scattering intensity multiplied by *q*. The difference scattering intensity, Δ*S*(*q*), is scaled by the number of solvent molecules and has the unit of electron units (e.u.).

First, we checked the validity of the *S-cube* simulation by comparing simulated difference scattering curves from *S-cube* for a model reaction and actual experimental data from synchrotrons and XFELs. For the model reaction, the photolysis of iodo­form (CHI_3_) in cyclo­hexane, where the experimental data corresponding to the reaction were previously analyzed and reported by our group (Ahn *et al.*, 2018 ▸), was selected. According to the study, there are two parallel reaction channels that contribute to the reaction intermediates at a time delay of 100 ps, which are the isomer channel (from the excited CHI_3_ to the CHI_2_I isomer) and the radical channel (from the excited CHI_3_ to the CHI_2_ and I radicals) with the molar fraction of the two intermediates being 40:60 at the time delay [as shown in the inset of Fig. 4(*e*) ▸].

For comparison, we simulated the averaged difference scattering curves corresponding to the model reaction while varying the number of difference scattering curves for averaging, using the same experimental parameters used in the experiment and with σ_solvent_ as obtained from an earlier study (Ahn *et al.*, 2018 ▸). The difference curves contain all relevant contributions including the heating signal (the solvent-only term) although the heating signal for this case with cyclo­hexane as solvent is negligible in the displayed *q* range. Fig. 4 ▸ compares the simulated and experimental difference scattering curves. The experimental data, obtained at a synchrotron, and the simulated data are generally consistent regardless of the number of difference curves. For a proper comparison, the experimental data are scaled to the simulated data using a scaling factor which is determined by following the scheme depicted in Figs. S3 and S4. The level of the residual (blue line), which is the deviation of the measured (or simulated) data from the corresponding expected values and which thereby represents the experimental noise of the experimental data, is quite well reproduced by the simulated data. The overall consistency of the residuals from the experiment and the simulation confirms that *S-cube* can satisfactorily simulate the degree of experimental noise.

**Figure 5:**
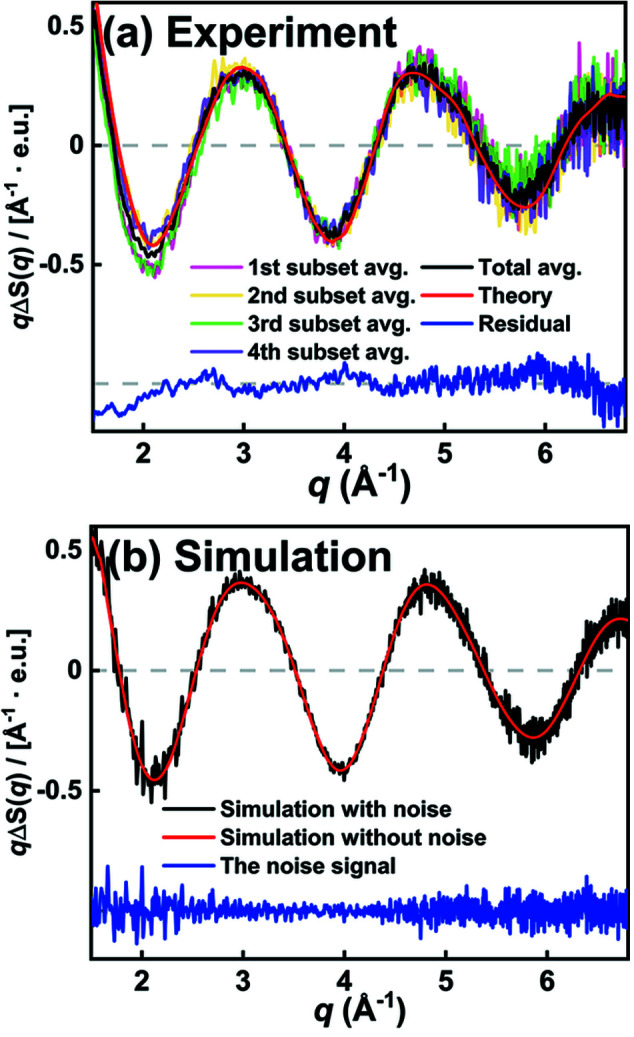
Comparison of (*a*) experimental TRXL data and (*b*) *S-cube* simulation for iodo­form in cyclo­hexane at a time delay of 100 ps after excitation at 267 nm, which was measured at the XSS beamline of PAL-XFEL. In (*a*), the black curve is the averaged difference scattering curve from 460 difference scattering curves, the red curve is the smoothed difference scattering curve from the averaged difference scattering curve, the blue curve is the residual between the two curves, and the transparent pink, yellow, green and purple curves are the averaged curves from subsets of 460 difference scattering curves, each of which consists of 115 difference scattering curves. In (*b*), the black, red and blue curves are simulation results with noise, simulation results without noise, and the residuals between simulation results with and without noise, respectively. The residuals in (*a*) and (*b*) represent noise. The *y*-axis indicates *q*Δ*S*(*q*), the difference scattering intensity multiplied by *q*. The difference scattering intensity, Δ*S*(*q*), is scaled by the number of solvent molecules and has the unit of electron units (e.u.).

To verify the applicability of *S-cube* to experiments at XFELs, we measured and used the experimental data corresponding to the same model reaction at PAL-XFEL (Kang *et al.*, 2017 ▸) for comparison with the *S-cube* data. σ_solvent_(*q*) was also measured in the same experiment, and it was used for the *S-cube* simulation in the experiment. The resulting experimental and simulated data show excellent agreement, as shown in Fig. 5 ▸. In Fig. 5(*a*) ▸, the average of 460 experimental difference scattering curves is shown together with the corresponding residual representing the noise level of the averaged experimental curve. More detailed experimental and simulated parameters are as follows. *f*: 1 kHz; *D*: 1.5 s; *n*: 5 × 10^8^ for Figs. 5(*a*)–5(*f*) ▸. Measurement times: 0.0008 h for Fig. 4(*b*) ▸, 0.1288 h for Fig. 5(*d*) ▸. The same *q* bins are used for both experimental and simulated data. For comparison, the simulated difference scattering curve and related noise level are depicted in Fig. 5(*b*) ▸. The amplitude of the simulated noise deviates from the experimental values at around *q* = 2.0 Å^−1^. To verify the origin of the discrepancy, we calculated the average curves of four different subsets of the experimental data, each of which consists of 115 experimental difference scattering curves [partial averages, shown in Fig. 5(*a*) ▸]. Although the partial averages show reasonable agreement overall, a noticeable inconsistency between the partial averages can be observed at around *q* = 2.0 Å^−1^. The deviation of each partial average from the overall average shows a tendency such that the deviation is either all positive or all negative at *q* = 2.0 Å^−1^ and adjacent *q*-points. This tendency of the fluctuation of difference scattering intensities in *q*-space is in clear contrast to the expected behavior of the quantum noise, which should randomly fluctuate between positive and negative around zero. Such correlations in the fluctuation of the signal in the adjacent *q*-points indicate that there is another noise source which contributes to the regular fluctuation of the signal, other than the quantum noise from the detector. We attribute the other source of the signal fluctuation to systematic noise arising from fluctuations in the experimental conditions, such as the thickness of the liquid jet or the intensity of the X-ray pulse (Haldrup, 2014 ▸; Ki *et al.*, 2019 ▸; van Driel *et al.*, 2015 ▸).

As manifested by the fluctuations of the partial averages, the difference scattering curves measured from the experiment inevitably are contaminated by systematic noise unless the experiment is performed in an ideally constant condition.

Accordingly, σ_solvent_(*q*) contains quantum noise and systematic noise from the fluctuations of the experimental conditions. As a result, if σ_solvent_(*q*) is used to represent the quantum noise alone, it would overestimate the quantum noise. We do not consider this overestimation to be a major problem because it would set the upper limit for the worst case, reflecting the potential systematic noise. Moreover, Fig. 5 ▸ shows that the noise in the simulation and experiment converges as the number of averaged curves increases.

When conducting a TRXL experiment, the diffraction signal from the sample can be either separately collected for each X-ray pulse in a shot-by-shot manner or accumulated for multiple X-ray pulses in an integration mode. The difference of the noise level depending on the two experimental modes can be inferred from the comparison between the standard deviation of experimental data measured at ESRF and PAL-XFEL (blue and magenta curves in Fig. S2, respectively), obtained by the integration and shot-by-shot schemes, respectively. Compared with the images measured in the integration mode, the images measured in the shot-by-shot manner in general display higher fluctuation of scattering intensities. There are two main reasons for this. One is that when the signal from multiple X-ray pulses are accumulated in an image, the effect of the fluctuation of the experimental conditions such as the X-ray pulse intensities and the thickness of the liquid jet are averaged over the period of accumulation. The other reason is that the overall electronic readout noise is higher for the shot-by-shot mode when normalized by the data collection time because the electronic readout noise per image is about the same.

### 2.3. Simulation of time-resolved curves   

A typical TRXL experiment yields time-resolved data measured at a number of time delays. The concentrations of reaction intermediates change over time delays, and a kinetic analysis of time-resolved data can extract such information and species-associated difference curves, which are often subject to further structural analyses. In this regard, it is desirable to simulate time-resolved difference curves as a function of the time delays. One of the most important parameters to be determined for a TRXL experiment is the number of time delays to cover the desired time range for a given measurement time, as the number of time delays during a limited measurement time exists in a trade-off relationship with the quality of the data, that is, the SNR of the data at each time delay in the *q*-space. As both a sufficient number of time delays and the quality of the data are prerequisites for the retrieval of reliable kinetics and structural changes, determining the optimal number of time delay points for a given measurement time plays an important role in a successful experiment.

**Figure 6:**
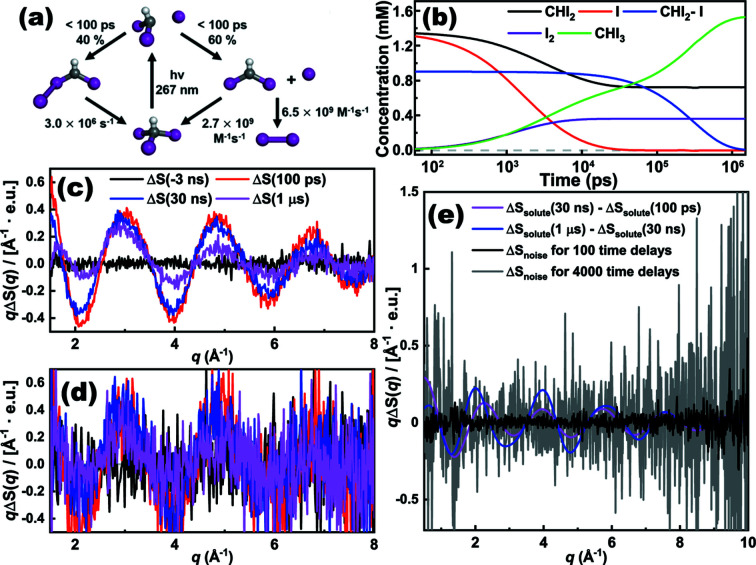
TRXL data simulation by *S-cube* for the photolysis reaction of CHI_3_. (*a*) Kinetics scheme used for the simulation. (*b*) Time-dependent concentrations (solid lines) according to the kinetics. (*c*, *d*) Time-resolved difference curves simulated assuming that the data are collected for 24 h of beam time at a synchrotron (ESRF) with 70% of duty cycle. The curves at four selected time delays, −3 ns, 100 ps, 30 ns and 1 µs, are shown among the entire series consisting of (*c*) 100 or (*d*) 4000 time delays. (*e*) Comparison of the noise level of the experimental data for (*c*) and (*d*) and the difference between the data at different time delays without any consideration of experimental noise. The *y*-axis for (*c*, *d*, *e*) indicates *q*Δ*S*(*q*), the difference scattering intensity multiplied by *q*. The difference scattering intensity, Δ*S*(*q*), is scaled by the number of solvent molecules and has the unit of electron units (e.u.).

For such a purpose, *S-cube* allows the user to estimate the quality of the experimental data from a given set of experimental parameters such as the duration of the beam time, the duty cycle and the number of time delays, and thereby facilitates the determination of the optimal number of time delays to be measured. As a demonstrative example, we simulated the TRXL data using *S-cube* for the photodissociation reaction of CHI_3_ for different numbers of time delays. The reaction scheme and the time-dependent concentrations of intermediates, which were reported from the previous TRXL study on the reaction, are shown in Fig. 6 ▸. Based on the time-dependent concentration profile, we simulated the experimental curves for two different number of time delays. For the simulation parameters, 24 h of beam time and 70% of duty cycle were assumed. In addition, it was assumed that the measurement time is the same for each time delay. The other parameters such as σ_solvent_(*q*) and the intensity of the X-ray pulses were set to be the same as in the simulations shown in Fig. 4 ▸. Fig. 6(*c*) ▸ shows the quality of the experimental data for four selected time delays, −3 ns, 100 ps, 30 ns and 1 µs, when the data are measured for a hundred time delays. It can be seen that the quality of the experimental data is sufficient enough to resolve the small changes in the shape of the signal. By contrast, in the case when the data are measured for 4000 time delays, the SNR of the data becomes much worse so that it is difficult to discern the differences between the experimental data for different time delays [see Fig. 6(*d*) ▸]. The noise level of the experimental data for two different numbers of time delays and the difference between the data for different time delays are shown in Fig. 6(*e*) ▸ for comparison. For 100 time delays, as the noise level of the data is much smaller than the difference between the experimental data, the change of the shape of the signal can be clearly resolved which eventually allows the change of the molecular structures during the reaction to be retrieved. However, when the data are measured at 4000 time delays for the same given measurement time, it is expected that such a change of the molecular structures would not be retrieved due to the low SNR of the experimental data. As shown through this demonstrative example, *S-cube* can be used to simulate TRXL data for a series of time delays with varying the number of time delays. Apparently, by inspecting the quality of the simulated TRXL data, one can determine the optimal number of time delays to achieve the goal of the experiment. Thus *S-cube* can be a useful tool for conducting a successful experiment within a given beam time.

### 2.4. Effect of heavy-atom labeling   

Because *S-cube* is a program that simulates TRXL data, we demonstrate the application of *S-cube* by simulating TRXL data for a series of target molecules that have photoinduced structural changes. Here, we selected the *cis*-*trans* photoisomerization of azo­benzene (AB) as a model of photoinduced structural change and simulated TRXL data corresponding to the structural change using *S-cube*. This reaction is one of the most representative photoisomerization reactions, and it has been studied for decades with various spectroscopic techniques (Bortolus & Monti, 1979 ▸; Schultz *et al.*, 2003 ▸; Stuart *et al.*, 2007 ▸). Despite the fact that *cis*-*trans* isomerization of AB has received much attention (Bandara & Burdette, 2012 ▸; Schultz *et al.*, 2003 ▸), it has been a challenge to examine the structural change during the reaction by using TRXL. The major obstacle during an investigation using TRXL is that AB consists of only light atoms, *i.e.* without any heavy atoms. As the scattering intensity from a molecule is proportional to the square of the number of electrons in the molecule, the weak scattering signal from AB makes it difficult to obtain the signal directly associated with the structural change of the molecule with a sufficient SNR. For the same reason, only a highly limited number of TRXL studies have been reported on molecules composed only of light atoms (Kim *et al.*, 2009 ▸; Leshchev *et al.*, 2018 ▸). Nevertheless, as an archetypal means of overcoming the low scattering cross section of such molecules, heavy-atom labeling using heavily scattering labels such as bromine with the molecules to enhance the scattering cross section has been proposed (Ihee, 2009 ▸; Ihee *et al.*, 2010 ▸; Ki *et al.*, 2017 ▸; Mathew-Fenn *et al.*, 2008 ▸). Using *S-cube*, we quantitatively evaluated how the heavy-atom labeling enhances the TRXL signal for the photoisomerization of AB. For this purpose, we also examined the TRXL signal corresponding to the photoisomerization of a di-bromo derivative of AB, 4,4′-di­bromo azo­benzene (Br_2_AB).

**Figure 7:**
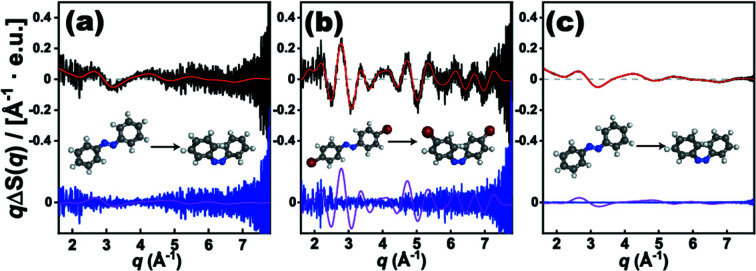
Comparison of *S-cube* simulation of photoisomerization of azo­benzene (AB) at PAL-XFEL and LCLS-II HE and 4,4′-di­bromo azo­benzene (Br_2_AB) at PAL-XFEL. The black, red and blue curves are simulation results with noise, simulation results without noise, and the residuals between simulation results with and without noise, respectively, and the magenta lines are solute-only signals. (*a*) *S-cube* simulation of photoisomerization of AB in cyclo­hexane at PAL-XFEL with accumulation time of 60 s and repetition rate (*f*) of 30 Hz, which corresponds to 540 difference scattering curves. (*b*) *S-cube* simulation of photoisomerization of Br_2_AB in cyclo­hexane at PAL-XFEL with accumulation time of 60 s and *f* of 30 Hz, which corresponds to 540 difference scattering curves. (*c*) *S-cube* simulation of photoisomerization of AB in cyclo­hexane at LCLS-II HE with accumulation time of 60 s, *f* of 1 MHz, which corresponds to 18000000 difference scattering curves. σ_solvent_(*q*) at LCLS-II HE is scaled from σ_solvent_(*q*) at PAL-XFEL with each value of photons per curve which are 3 × 10^10^ for LCLS-II HE and 1 × 10^12^ for PAL-XFEL. The *y*-axis indicates *q*Δ*S*(*q*), the difference scattering intensity multiplied by *q*. The difference scattering intensity, Δ*S*(*q*), is scaled by the number of solvent molecules and has the unit of electron units (e.u.).

As shown in Fig. 7(*a*) ▸, using *S-cube*, we simulated the TRXL signal arising from the photoisomerization reaction of 50 m*M* AB in cyclo­hexane with a 10% excitation ratio. In Fig. 7(*b*) ▸, we also simulated the TRXL signal for the photoisomerization reaction of Br_2_AB under the same experimental conditions. To examine the effect of the heavy-atom labeling on the TRXL signal in terms of the noise level, we ignored structural differences other than the existence of heavy atoms between AB and Br_2_AB by simply replacing two H atoms with two Br atoms in the structure of AB [see Fig. 7(*b*) ▸]. For the simulations shown in Figs. 7(*a*) and 7(*b*) ▸, σ_solvent_(*q*) at the XSS beamline of PAL-XFEL was used.

Fig. 7(*a*) ▸ shows the TRXL signal simulation results of the photoisomerization of AB for PAL-XFEL. The solute signal is much lower than the noise estimated from σ_solvent_(*q*) in PAL-XFEL, indicating that the experiment would yield data sufficient for elucidating the molecular structural change occurring during the reaction. On the other hand, Fig. 7(*b*) ▸, showing the simulation result of the photoisomerization of the heavy atom substituent Br_2_AB, indicates that the solute signal is dominant compared with the noise. Therefore, it can be seen from the comparison of Figs. 7(*a*) and 7(*b*) ▸ that the heavy-atom labeling clearly offers great potential to observe structural changes at PAL-XFEL.

### 2.5. The prospect of LCLS-II HE   

Although heavy-atom labeling of molecules can be an effective means by which to overcome the low scattering signals arising from molecules consisting of only light atoms (Andersson *et al.*, 2008 ▸; Malmerberg *et al.*, 2011 ▸), one cannot completely rule out the possibility of unexpected structural distortion due to the attached heavy atoms. Therefore, it remains desirable to obtain the signal from an intact molecule without heavy-atom labeling. In this regard, we considered the possibility of obtaining TRXL data without heavy-atom labeling in the future XFELs as the performance of state-of-the-art XFEL is evolving remarkably. One typical example showing the evolution of the performance of XFEL is LCLS-II HE (Schoenlein *et al.*, 2017 ▸), which is expected to have an *f* value of 1 MHz, representing a substantial improvement by a factor of tens of thousands compared with PAL-XFEL with a minute loss of *n* by a factor of dozens (Kim, Kim *et al.*, 2018 ▸). In terms of *N*, there would be an improvement by a factor of several thousand, which would eventually lead to a significant improvement of the SNR of the experimental signal. Accordingly, it is expected that the evolution of the performance of the XFELs would open up new experimental possibilities, such as the ability to capture the small signals that could not be resolved at currently available XFELs. Hence, we suggest that *S-cube* can be utilized to quantitatively predict the quality of future experimental data which can be obtained using these XFELs at higher performance levels. To demonstrate this aspect, we estimated the experimental signal corresponding to the photoisomerization of AB using *S-cube* for experiments at one of the representative future XFELs, LCLS-II HE. The following two assumptions applied when estimating Δ*S*_noise_(*q*) from the experiment at LCLS-II HE. The first is that σ_solvent_(*q*) is proportional to the square root of *N*. By using this assumption, we estimated σ_target_(*q*) at LCLS-II HE from σ_solvent_(*q*) at PAL-XFEL using the following formula, which is closely related to equation (4),
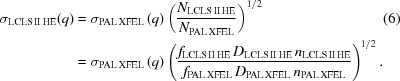
The second assumption is that σ_target_(*q*) stems solely from the quantum noise. However, it should be noted that there are other sources of noise as well, *e.g.* systematic noise due to fluctuations of the experimental conditions, that contribute to σ_target_(*q*). As demonstrated in Fig. 5 ▸, the presence of such systematic noise makes it difficult for the second assumption to be strictly valid when estimating actual experimental data. Nevertheless, as discussed in Fig. 5 ▸, even an estimation under a flawed assumption still allows a reasonable prediction of the feasibility of certain ground-breaking experiments at new X-ray source facilities, as the noise level in *S-cube* is purposely overestimated under this assumption and thus can be regarded as able to provide an upper limit in terms of the noise level.

For the simulation shown in Fig. 7(*c*) ▸, the σ_solvent_ used for the simulations, of which the results are shown in Fig. 7 ▸, indeed cannot be directly measured from LCLS-II HE, but estimated from experimental data measured at PAL-XFEL. For the estimation, σ_solvent_ from PAL-XFEL was simply scaled on the basis of the different number of incident photons. The *n* values at PAL-XFEL and LCLS-II HE were set to 1 × 10^12^ and 3 × 10^10^, respectively, and the corresponding *f* values were considered to be 30 Hz and 1 MHz (Kim, Kim *et al.*, 2018 ▸). An accumulation time of 36 s was used in both simulations for the PAL-XFEL and LCLS-II HE cases, after which the overall 540 and 1.8 × 10^7^ difference scattering curves were averaged to yield the resulting simulated difference scattering curves. Fig. 7(*c*) ▸ shows the result of the simulation of the experiment at LCLS-II HE. When the result is compared with that from PAL-XFEL, despite the fact that the measurement times for both simulated data are identical, there is a considerable difference in the SNR of the data. The curve obtained from LCLS-II HE shows a much clearer signal than that from PAL-XFEL due to the large value of *f* of LCLS-II HE (1 MHz). The quantitative SNR estimation by *S-cube* indicates that the improved performance of XFEL will allow the resolution of structural changes of molecules with light atoms, such as the photoisomerization of AB.

## 3. Conclusions   

In this work, we introduce *S-cube*, which can simulate the solute-only signal, the solute–solvent cross signal, the solvent-only signal and the noise for a target reaction, a desired solvent, a target beamline and a given data collection time. The purpose of *S-cube* is to allow the user to calculate the expected noise level routinely compared with the scattering signal, the relative magnitude of the solute-only signal against the solvent-only signal and the simplified cage signal using the hard-spheres approximation. We expect that *S-cube* well help in the design of successful TRXL experiments at both synchrotrons and XFEL beamlines.

## 4. Distribution   

*S-cube* is an app that can be used in MATLAB for free. The *S-cube* program (doi:10.5281/zenodo.3637919) is distributed in https://zenodo.org/badge/latestdoi/207274974 as well as the GitHub repository (https://github.com/Jkim9486/Scube).

## 5. Methods   

### 5.1. Detailed procedure of the TRXL data simulation using *S-cube* and its theoretical background   

For randomly oriented molecules, the X-ray scattering intensity from chemical species (reactants, intermediates and products) can be calculated by the Debye equation using the molecular structures of chemical species,
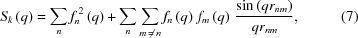
where *q* is the momentum transfer vector between the incident and elastically scattered X-ray waves, *S*_*k*_(*q*) is the X-ray scattering intensity for the chemical species *k*, the indices *m* and *n* include all atoms in the chemical species, *r*_*nm*_ is the distance between the *n*th and *m*th atoms, and *f*_*n*_(*q*) and *f*_*m*_(*q*) are correspondingly the atomic form factors of the *n*-type atoms and *m*-type atoms.

The solute-only signal is one of the components of the TRXL signal stemming from the structural changes of the solute molecules. Some of the reactant molecules excited by the pump pulse transform into intermediates or products. The associated changes in the intramolecular atomic coordination affect the solute-only signal, which can be calculated according to the following equation,
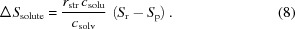
Here *c*_solu_ is the concentration of the solute; *c*_solv_ is the concentration of the solvent, which is used to scale the amplitude of the solute-only signal to one mole of the solvent molecules; *S*_r_(*q*) and *S*_p_(*q*) are scattering intensities of the reactant and product solute molecules, respectively, which are calculated from the atomic coordinates of the reactants and products using the Debye equation; and *r*_str_ is the ratio of the solute molecules that are converted to the product through the reaction to the total number of solute molecules.

The solute–solvent cross signal, which is also called the cage signal, results from the interference between the atoms in the solute molecules and those in the solvent molecules. Therefore, the solute–solvent cross signal is sensitive to structural changes of the solvation cage surrounding the solute molecules [see Fig. 1(*b*) ▸]. Typically, the solute–solvent cross signal is obtained from the sine-Fourier transform of pair distribution functions, which can be calculated from MD simulations (see Fig. S3). For the TRXL simulation, it is necessary to obtain the solute–solvent cross signal from the MD simulations for every solute structure. On the other hand, because the scattering intensity is proportional to the square of the electron of the atom, the solute–solvent cross signal becomes negligible if the solute molecule is heavy enough. Because MD simulations for obtaining the solute–solvent cross signal require a large amount of calculation power compared with the other three signals and given that the purpose of *S-cube* is not to provide an accurate theoretical curve but a rapid estimation of the expected signal level relative to the noise level, we decided to use an approximation method rather than relying on the time-consuming MD simulations. Specifically, *S-cube* uses simplified pair distribution functions, which are approximated by trapezoidal functions. More specifically, the pair distribution function between the pair of two different elements *A* and *B* was approximated as a trapezoidal function of which the value is zero for distances shorter than the sum of the van der Waals radius of *A* and *B* and is equal to unity for distances longer than the sum of the van der Waals radius of *A* and *B* and the size of the solute molecule, *r*_s_, increasing linearly for distances between the two. The size of the solute molecule, *r*_s_, was approximated using the following formulae,
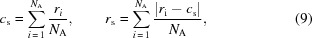
where *c*_s_ is the center of the solute molecule, *r*_*i*_ is the atomic coordinates of the *i*th atom in the solute molecule, *N*_A_ is the number of atoms in the solute molecule and |*r*_*i*_ − *c*_s_| is the distance of the *i*th atom in the solute molecule from the center of the molecule. Equation (9) is similar to the formula for obtaining the radius of gyration of a molecule but is different in that the contribution of each atom by its atomic mass is ignored in order to focus solely on the distribution of the positions of the atoms.

The solvent-only signal arises from the changes in the temperature and density of the solvent which originate from the heat released from the light-absorbing solute molecule. The solvent-only signal can be expressed by the following equation,
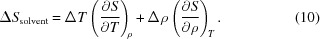
In equation (10), the two differentials (δ*S*/δ*T*)_ρ_ and (δ*S*/δρ)_*T*_ or commonly employed solvents such as aceto­nitrile, cyclo­hexane, methanol, ethanol, di­chloro­methane chloro­form and carbon tetrachloride are well documented in the literature (Kim *et al.*, 2009 ▸; Cammarata *et al.*, 2006 ▸). For calculation of the solvent signal, maximum changes of temperature (Δ*T*) and density (Δρ) are calculated from the energy of the pump laser and the number of light-absorbing solute molecules by assuming that all energy absorbed by the excited molecules (*Q*_max_) is transferred as heat to the solvent without energy loss. This assumption is not strictly accurate, but provides the maximum amount of *Q* to avoid underestimating the solvent signal,
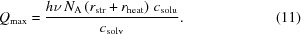
Equation (11) is the maximum heat transformed from the excited solute to the solvent where, *h* (J s^−1^) is Planck’s constant, ν (s^−1^) is the frequency of the pump laser, *N*_A_ is Avogadro’s number, *r*_heat_ is the ratio of the excited solute molecules that do not undergo a subsequent structural transition and only release the absorbed energy as heat relative to the total number of excited solute molecules. The maximum temperature and density changes, Δ*T*_max_ and Δ*ρ*_max_, are obtained from *Q*_max_ assuming isochoric and isobaric processes, respectively, via the following equations,
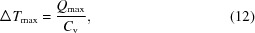


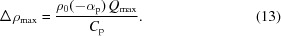
In equations (12) and (13), *C*_v_ (J mol^−1^ K^−1^) and *C*_p_ (J mol^−1^ K^−1^) are the heat capacities at a constant volume and pressure, respectively; α_p_ (K^−1^) is the volumetric thermal expansion coefficient, also known as the isobaric dilation constant; and *ρ*_0_ is the density of the solvent. The time dependence of the solvent-only signal in equation (10) can be precisely calculated if the energy levels of all intermediates and products are provided. For a fast calculation, as an approximation, Δ*T* is set to Δ*T*_max_ up to 10 ns and to decrease linearly to reach zero by 3 µs, while Δρ is set to zero up to 10 ns and is set to increase linearly to Δ*ρ*_max_ by 3 µs.

The scattering intensity of the solution is represented by the sum of the solute-only, solute–solvent cross and solvent-only signals in the following equation,
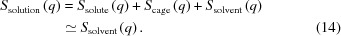
In this equation, *S*_solution_(*q*) denotes the total scattering intensity from the solution and *S*_solute_(*q*), *S*_cage_(*q*) and *S*_solvent_(*q*) denote the components of the total scattering intensities which originate from the contributions of the solute, the solvent molecules (cage) surrounding the solute molecule and the bulk solvent, respectively. In general, *S*_solvent_(*q*) dominates the signal. Because the variance of the scattering intensity is proportional to the scattering intensity TRXL simulation, it can be expressed as follows (Kim *et al.*, 2009 ▸),
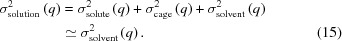
In equation (15), σ_*k*_ is the standard deviation of *S*_*k*_(*q*). To simplify the estimation, the total standard deviation, σ_solution_(*q*), can be approximated to be equal to σ_solvent_(*q*) because the number of solvent molecules is high enough to neglect the other two contributions from the solute molecules and cages, σ_solute_(*q*) and σ_cage_(*q*), respectively. In other words, regardless of the type of solute, σ_sol_(*q*) can be approximated by σ_solvent_(*q*) which can be measured in a separate experiment on a neat solvent.

## 6. Related literature   

The following references, not cited in the main body of the paper, have been cited in the supporting information: Wulff *et al.* (2003 ▸, 2007 ▸).

## Supplementary Material

Sections S1 to S3 and Figures S1 to S5. DOI: 10.1107/S1600577520002738/yi5089sup1.pdf
