# Supplementary material for: Estimating signal and noise of time-resolved X-ray solution scattering data at synchrotrons and XFELs
Source: J Synchrotron Radiat. 2020 Mar 31;27(Pt 3):633–45. doi: 10.1107/S1600577520002738 (PMC7206544; doi:10.1107/S1600577520002738)
Supplement: Supplementary file 1 [file s-27-00633-sup1.pdf]

Supporting information

S1. Supplementary Methods

S1.1. TRXL experiment of CHI_3 in cyclohexane at PAL-XFEL

The TRXL experiment was conducted at the XSS-FXL beamline of Pohang Accelerator Laboratory x-ray Free-electron Laser (PAL-XFEL). A typical setup for TRXL was used in the experiment. A femtosecond laser pulse from a Ti:sapphire laser was frequency-tripled to 267 nm and used to initiate the reaction. The laser pulse was focused to a spot of 300 um diameter to give 2.05 mJ/mm2 fluence on the sample. Subsequently, a femtosecond x-ray pulse generated at PAL-XFEL was used to probe the progress of the reaction. The x-ray pulse had a center energy of 12.7 keV and was focused to a spot of 30 um diameter on the sample. The pulse repetition rate (f) of the 267 nm laser pump pulse and the x-ray probe pulse are 15 and 30 Hz, respectively. The 2D scattering images were detected by the Rayonix MX225-HS charge-coupled-device detector (4096×4096 pixels, 15 μm pixel size) with 4×4 binning and azimuthally integrated into 1D difference scattering curves. The difference scattering images were collected with the 100 ps time delay between the laser pump and x-ray probe. For the sample, a 20 mM solution of iodoform (CHI_3) in cyclohexane was used. An open-jet system with a quartz capillary nozzle with the inner diameter of 100 μm was used to continuously provide a stable liquid jet of the sample.

S1.2. Consideration of signal-to-noise ratio in S-cube

According to Sedlak, S. M *et al.* (Sedlak *et al.*, 2017), if we consider the quantum noise as the only source of noise of the signal, the relation between the number of pixels belonging to the same q bin, which changes as the sample-to-detector distance changes, and the noise level of the q bin can be expressed as follows:

$$\sigma^2(q) = \frac{\sum_{i=1}^{M(q)} n_i}{M(q)^2} = \frac{N(q)}{M(q)^2} \tag{1}$$

where σ(q) is the standard deviation of scattering intensity, $M(q)$ is the number of pixels belonging to the q bin, and n_i is the number of counts per pixel belonging to the q bin, $N(q)$ is the sum of n_i. Following Eqn. S1, the signal-to-noise ratio (SNR) of the q bin can be derived as follows:

$$SNR = \frac{\sigma(q)}{I(q)} = \frac{\sqrt{N(q)}}{N(q)} = \sqrt{N(q)} \tag{2}$$

where I(q) is scattering intensity, that is, $N(q)/M(q)$. Eqn. S2infers that the SNR of the q bin is independent of the number of pixels belonging to the q bin. In other words, the SNR is not affected by the sample-to-detector distance. Instead, the SNR is dependent on the sum of counts per pixel

IMPORTANT: this document contains embedded data - to preserve data integrity, please ensure where possible that the IUCr Word tools (available from http://journals.iucr.org/services/docxtemplate/) are installed when editing this document.

belonging to the q bin. Thus, in this model system, the SNR is independent of experimental parameters such as sample to detector distance.

S1.3. Consideration of the maximum q-value distance in S-cube

In S-cube, the maximum q-value for the sample-to-detector distance provided by the user is calculated using the following equations:

$$q_{max} = \frac{4\pi}{\lambda}\sin\theta_{max}\,,\theta_{max} = \sin^{-1}\left(\frac{\lambda q_{max}}{4\pi}\right) \quad (3)$$

$$d \times \tan 2\theta = d' \times \tan 2\theta_{max}'\,,\theta_{max}' = \frac{1}{2}\tan^{-1}\left(\frac{\tan 2\theta_{max}}{k}\right) \quad (4)$$

$$q_{max}' = \frac{4\pi}{\lambda}\sin\theta_{max}' \quad (5)$$

where q_{max} and q'_{max} are the maximum q-values for the reference solvent scattering data and the target data, respectively, d and d' are sample-to-detector distances for the reference solvent scattering data and the target data, respectively, θ_{max} and θ'_{max} are the maximum theta-value for the reference solvent scattering data and the target data, respectively, and λ is the wavelength of incident x-ray.

S2. Supplementary Figures

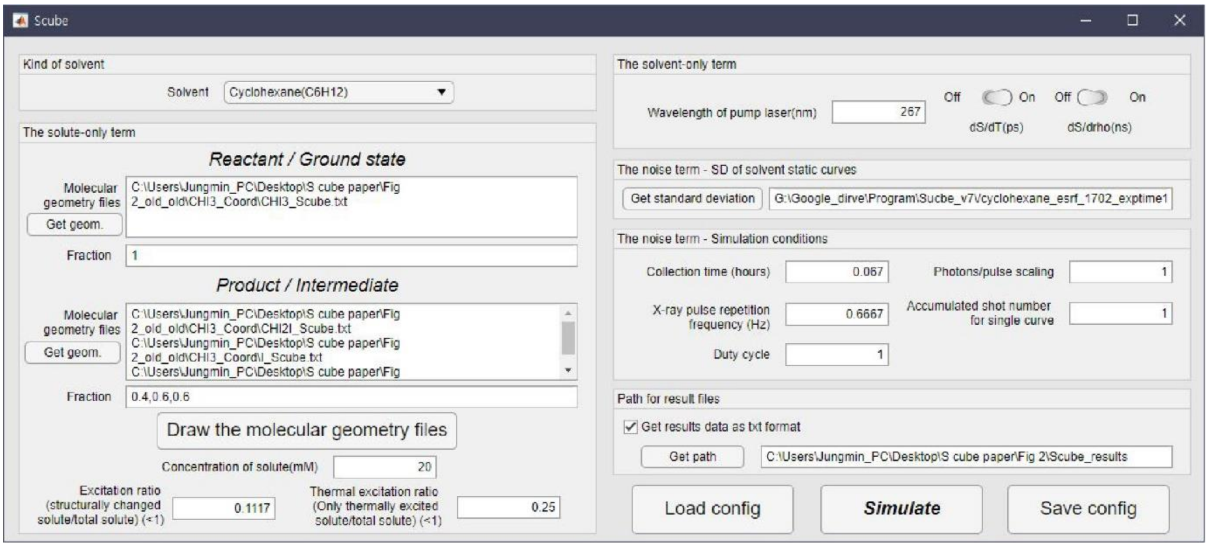

Figure S1 A snapshot of the graphical user interface of S-cube.

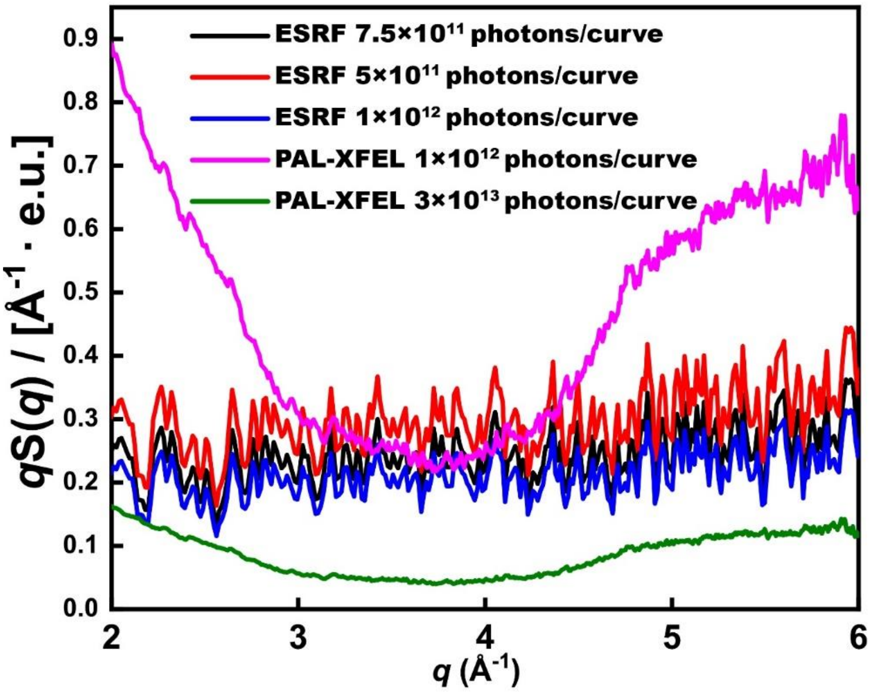

Figure S2 The standard deviation (SD) of scattering intensity, qS(q), for cyclohexane from the ESRF and PAL-XFEL scaled with target the number of incident scattering photons to the sample per scattering curve (N). The black and magenta curves are SD of the ESRF and PAL-XFEL measured from each experiment, respectively. The *n* of PAL-XFEL was roughly reported to ca. 1×10^{12}, and a scattering curve is obtained from the shot-by-shot measuring mode (*f : 30 Hz, D: 1/30 s*), the *N* of PAL-XFEL is assumed to $1x10^{12}$ (the black curve). *n* of the ESRF was reported to ca. 5×10^{8}, and a scattering curve is obtained from the accumulation mode with detector exposure time of 1.5 s and repetition rate of 1 kHz, *N* of ESRF is assumed to 7.5×10^{11} (the magenta curve) (Wulff *et al.*, 2007; Wulff *et al.*, 2003). The green curve is SD of the PAL-XFEL scaled to have *N* of 3×10^{13}. By comparing the blue (ESRF, photons per curves of 1×10^{12}) and magenta (PAL-XFEL, photons per curve of 1×10^{12}) curves, it suggests that the scaling between the SD from different experiments is not sophisticated, but scaling by one order of magnitude is possible. By comparing the red (ESRF, N of 5×10^{11}) and green (PAL-XFEL, N of 3×10^{13}) curves equally pretended to *d* of 1 second, it suggests that the scaled SD with same *D* of the XFEL is relatively smaller than that of ESRF. The SD of cyclohexane scattering intensities show distinct trends in *q*-space depending on the experimental facility. We suggest the reason for the distinct trends is the difference in the experimental setup including the stability of the x-ray photon intensity, the shape and size of the detector, the distance between the detector and the liquid jet, and the stability of the liquid jet. The y-axis indicates $q\Delta S(q)$, the difference scattering intensity multiplied by q. The difference scattering intensity, $\Delta S(q)$, is scaled by the number of solvent molecules and has the unit of electron unit (e.u.).

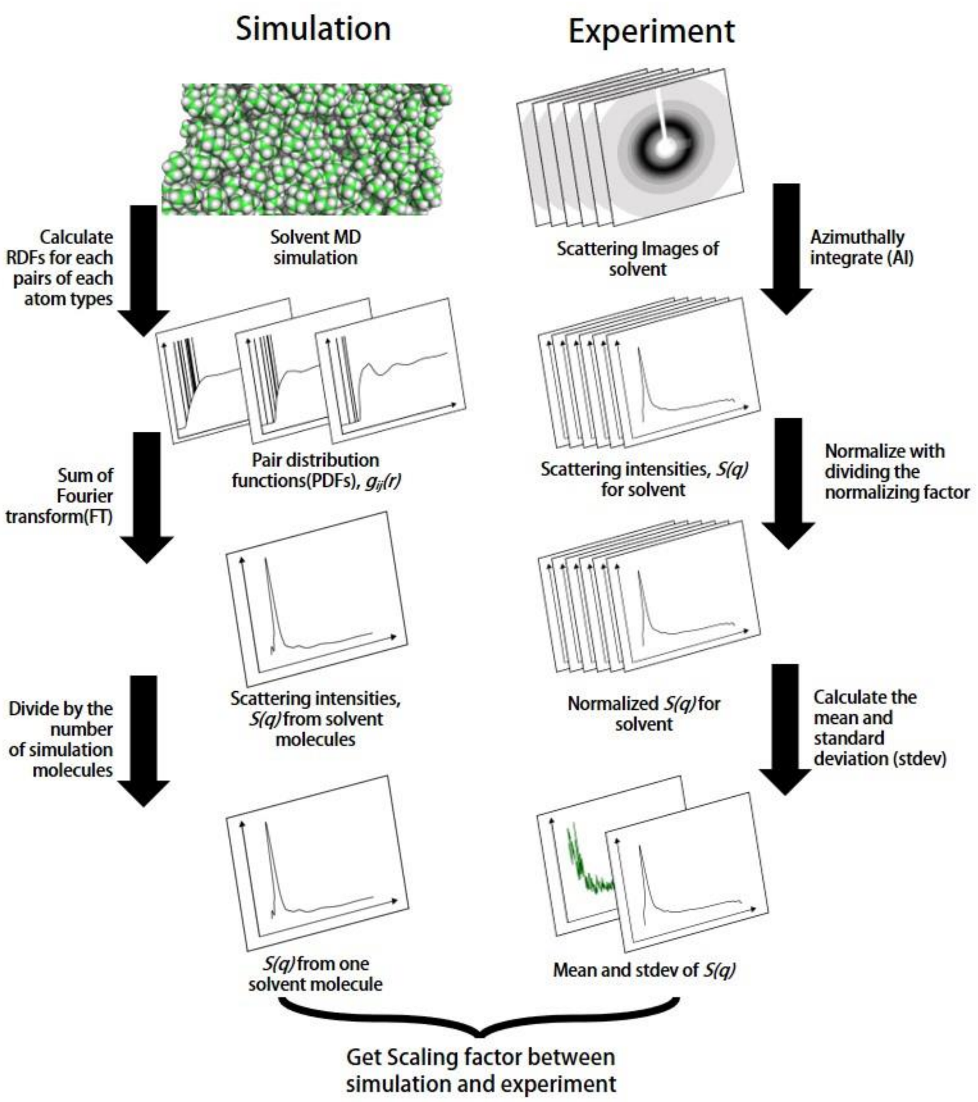

Figure S3 A scheme to obtain the scaling factor between the simulated curve (left column) and the experimental curve (right column). The simulated scattering intensity, $S_{sim}(q)$ is obtained from the sum of the sine Fourier transforms of radial distribution functions for each atom pair in the solvent molecule. $S_{sim}(q)$ is normalized to $S_{sim}(q)$ per one solvent molecule by dividing the former by the number of solvent molecules used in the MD simulation. Experimental 2D scattering images are azimuthally integrated to 1D scattering intensities. The 1D scattering intensities are normalized with the integral area of a specific, common q range. These normalized scattering intensities are scaled to the intensity of experiment, $S_{exp}(q)$ (See Figure S4). On the other hand, the standard deviation of $S_{exp}(q)$ is used to estimate the noise for S-cube simulation after scaling between simulation and experiment.

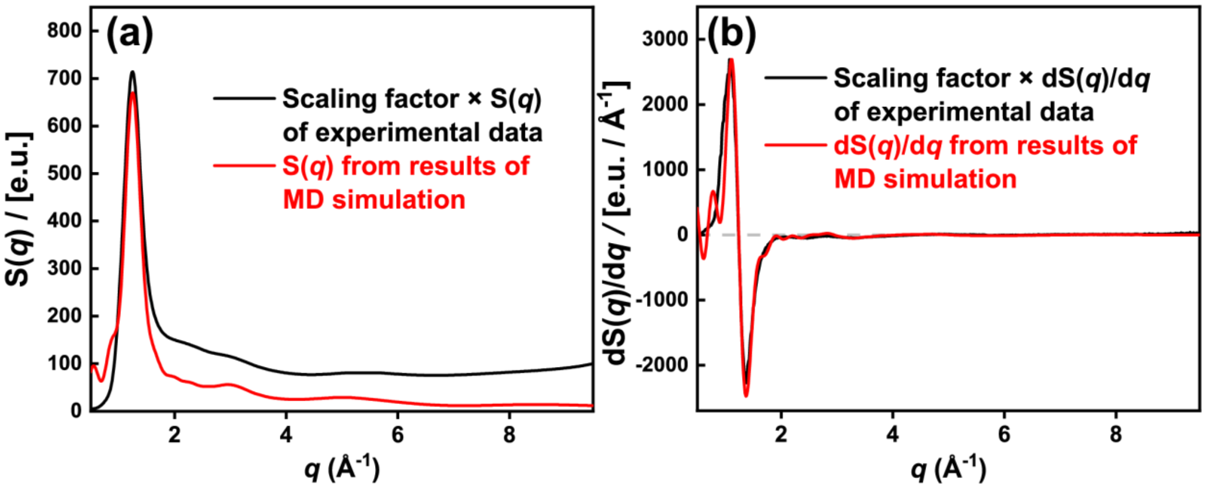

Figure S4 An example of determining the scaling factor between the experimental data (the black lines) and the simulated data calculated from the result of MD simulation (the red lines). (a) Comparison for scattering intensities, S(q) from MD simulation and experiment with a scaling factor. (b) Comparison for derivatives of S(q) from MD simulation and experiment with respect to q with a scaling factor. The difference scattering intensity, S(q), is scaled by the number of solvent molecules and has the unit of electron unit (e.u.).

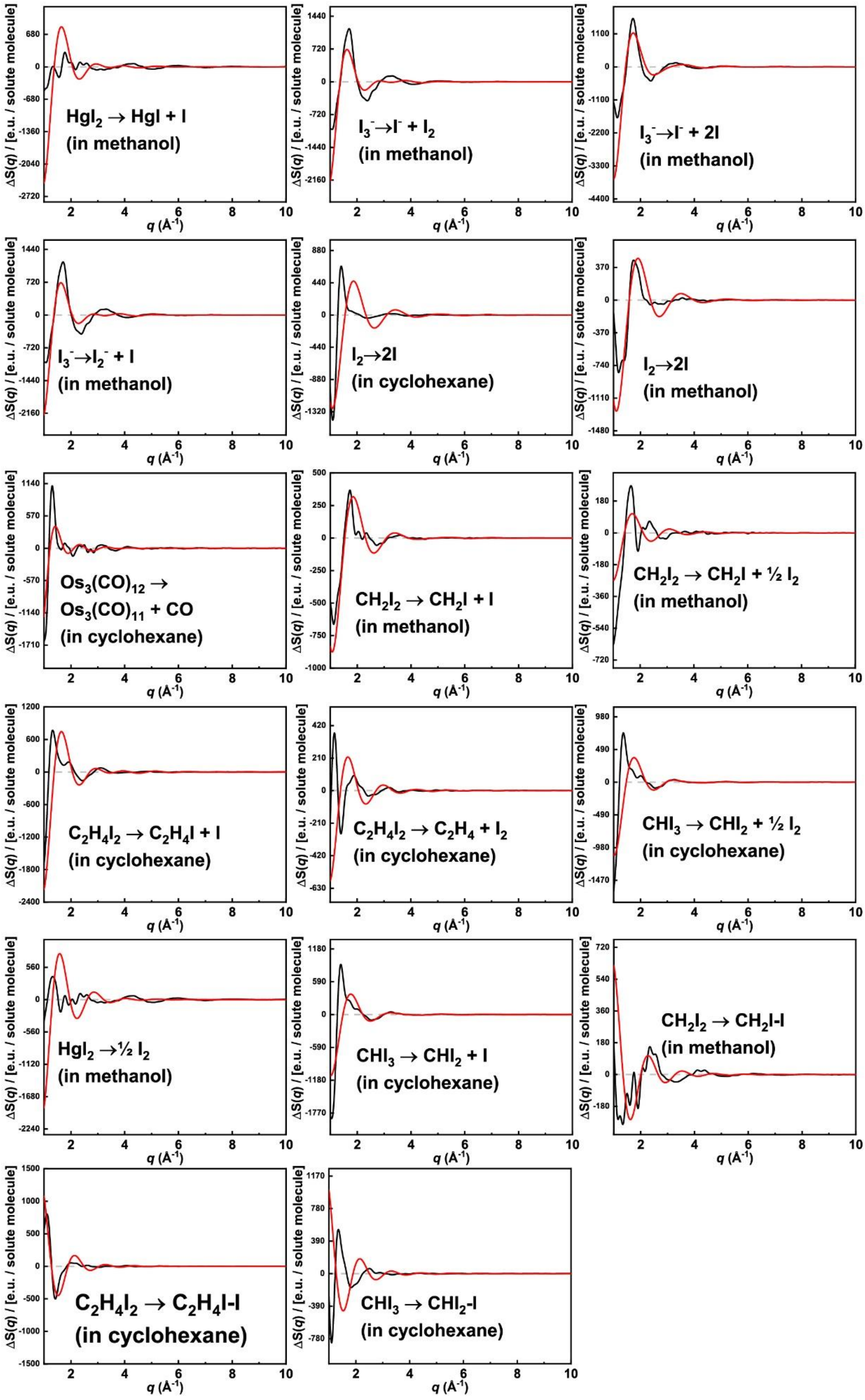
$\Delta S(q)$ / [e.u. / solute molecule]
q (Å^{-1})
$HgI_2 \rightarrow HgI + I$ (in methanol)
$I_3^- \rightarrow I^- + I_2$ (in methanol)
$I_3^- \rightarrow I^- + 2I$ (in methanol)
$I_3^- \rightarrow I_2^- + I$ (in methanol)
$I_2 \rightarrow 2I$ (in cyclohexane)
$I_2 \rightarrow 2I$ (in methanol)
$Os_3(CO)_{12} \rightarrow Os_3(CO)_{11} + CO$ (in cyclohexane)
$CH_2I_2 \rightarrow CH_2I + I$ (in methanol)
$CH_2I_2 \rightarrow CH_2I + ½ I_2$ (in methanol)
$C_2H_4I_2 \rightarrow C_2H_4I + I$ (in cyclohexane)
$C_2H_4I_2 \rightarrow C_2H_4 + I_2$ (in cyclohexane)
$CHI_3 \rightarrow CHI_2 + ½ I_2$ (in cyclohexane)
$HgI_2 \rightarrow ½ I_2$ (in methanol)
$CHI_3 \rightarrow CHI_2 + I$ (in cyclohexane)
$CH_2I_2 \rightarrow CH_2I\text{-}I$ (in methanol)
$C_2H_4I_2 \rightarrow C_2H_4I\text{-}I$ (in cyclohexane)
$CHI_3 \rightarrow CHI_2\text{-}I$ (in cyclohexane)

Figure S5 Comparison of solute-solvent cross terms obtained from MD simulations (black curves) and the hard-spheres approximation (red curves) for various reactions. The difference scattering intensity, ΔS(q), is for one solute molecule and divided by the scattering intensity of a single electron. As a result, ΔS(q) is in electron unit (e.u.) per solute molecule.

S3. Supplementary References

Sedlak, S. M., Bruetzel, L. K. & Lipfert, J. (2017). *J. Appl. Crystallogr.* **50**, 621-630.

Wulff, M., Kong, Q., Cammarata, M., Lo Russo, M., Anfinrud, P., Schotte, F., Lorenc, M., Ihee, H., Kim, T. K. & Plech, A. (2007). *AIP Conference Proceedings*, pp. 1187-1194. AIP.

Wulff, M., Plech, A., Eybert, L., Randler, R., Schotte, F. & Anfinrud, P. (2003). *Faraday Discuss.* **122**, 13-26.
